# *Ligusticum chuanxiong* Hort. Targets hsa-miR-10a-5p to Potentially Induce Apoptosis and Modulate Lipid Metabolism in Glioblastoma: A Natural-Product-Based Therapeutic Strategy

**DOI:** 10.3390/ph18101553

**Published:** 2025-10-15

**Authors:** Xiao-Xuan Cai, Hua-Li Zuo, Jing Li, Hsi-Yuan Huang, Li-Ping Li, Jie Ni, Pei-Sen Wu, Xiao-Yuan Xu, Dan Zhang, Yue-Yang Xie, Hsien-Da Huang, Yang-Chi-Dung Lin

**Affiliations:** 1 Warshel Institute for Computational Biology, School of Medicine, The Chinese University of Hong Kong, Shenzhen 518172, China; xiaoxuancai@link.cuhk.edu.cn (X.-X.C.); zuohuali@cuhk.edu.cn (H.-L.Z.); lijing0903@cuhk.edu.cn (J.L.); huanghsiyuan@cuhk.edu.cn (H.-Y.H.); liliping@cuhk.edu.cn (L.-P.L.); jennyni@cuhk.edu.cn (J.N.); yueyangxie@link.cuhk.edu.cn (Y.-Y.X.); 2 School of Medicine, The Chinese University of Hong Kong, Shenzhen 518172, China; peisenwu@link.cuhk.edu.cn (P.-S.W.); xiaoyuanxu@link.cuhk.edu.cn (X.-Y.X.); 3 Guangdong Provincial Key Laboratory of Digital Biology and Drug Development, The Chinese University of Hong Kong, Shenzhen 518172, China; 4 Longgang Central Hospital of Shenzhen, Shenzhen 518116, China; 13762470784@163.com; 5 Department of Endocrinology, Key Laboratory of Endocrinology of National Ministry of Health, Peking Union Medical College Hospital, Chinese Academy of Medical Sciences & Peking Union Medical College, Beijing 100730, China

**Keywords:** traditional Chinese medicine, glioblastoma (GBM), miRNA, metabolism, transcriptomics

## Abstract

**Background/Objectives:** Glioblastoma (GBM), the most aggressive primary malignant brain tumor, has a dismal prognosis and limited treatment options. The dried rhizome of *Ligusticum chuanxiong* Hort. (Chuanxiong, CX) is a traditional Chinese medicinal herb frequently prescribed in formulas intended to invigorate blood circulation. CX also exhibits anti-glioma activity, but its molecular mechanisms remain incompletely understood. **Methods:** In this study, we combined transcriptomics and Raman spectroscopy to investigate the effects of reconstituted CX-dispensing granules (hereafter referred to as CXG solution) on U87MG cells, suggesting their dual role in promoting cell death and modulating collagen deposition and lipid metabolism. **Results:** Mechanistically, we demonstrated that the CXG solution downregulates hsa-miR-10a-5p, which directly targets BCL2L11, known to induce pro-apoptotic effects, as validated by qPCR and dual-luciferase reporter assays. Furthermore, the CXG solution and hsa-miR-10a-5p suppress lipid metabolism through a coherent feed-forward loop via targeting transcription factors SREBF1 and E2F1. An electrophoretic mobility shift assay (EMSA) confirmed E2F1 binds to the hsa-miR-29a promoter, leading to the synergistic repression of hsa-miR-29a-3p by SREBF1 and E2F1. Network pharmacology analysis combined with molecular docking suggested that the ferulic acid and adenosine in CX potentially modulate EGFR-the E2F1-hsa-miR-10a-5p axis. **Conclusions:** These findings elucidate CX’s multi-target anti-GBM mechanisms and propose a novel therapeutic strategy combining metabolic intervention with miRNA-targeted therapy, providing novel insights into feed-forward loop regulation in miRNA networks.

## 1. Introduction

Glioblastoma (GBM) is the most common and most aggressive primary malignant tumor of the central nervous system, characterized by rapid progression, high invasiveness, and a high rate of recurrence. The overall 2-year survival rate of GBM patients is 18–33%, regardless of advanced treatment that includes maximal surgical resection, followed by radiotherapy plus concomitant and maintenance temozolomide chemotherapy [1,2,3]. One of the major challenges in GBM treatment lies in the development of therapeutic resistance and frequent tumor relapse. Emerging evidence suggests that metabolic reprogramming, particularly involving lipid metabolism [4] and extracellular matrix remodeling, such as collagen deposition, plays critical roles in tumor progression, therapy resistance, and poor prognosis [5].

Traditional Chinese medicine (TCM), with its multi-target and system-level therapeutic approach, has gained increasing attention for its potential in treating complex diseases, such as GBM, and those related to metabolic syndrome. Classified as spicy-warm and entering the liver, gallbladder, and pericardium meridians, CX disperses wind-cold and mobilizes both qi and blood; consequently, practitioners prescribe it alone or in classic formulas such as Chuanxiong Cha-Tiao-San for disorders ranging from migraines and cerebrovascular events to contusions, oedema, and menstrual cramps. Modern pharmacological studies reveal that extracts and purified constituents of the herb act on the brain, cardiovascular, hematological, and nervous systems, offering antioxidant, neuroprotective, anti-inflammatory, and analgesic effects that together highlight its pronounced adaptogenic potential [6,7]. It has also recently been explored for its anti-tumor effects in brain malignancies. Accumulating evidence suggests that CX and its hallmark constituents hinder glioma progression through multiple mechanisms [8]. Notably, CX is a key ingredient in the herbal formula “Pingliu Keli,” which has been reported to inhibit glioma cell proliferation and induce apoptosis [9]. Active compounds extracted from CX, such as tetramethylpyrazine, n-butylidenephthalide, ferulic acid, and ligustilide, have demonstrated the ability to suppress GBM growth by inhibiting proliferation, tumor migration, invasion, and sensitizing GBM to temozolomide treatment targeting CXCR4, Rho GTPases, PI3K/Akt, ERK1/2, and Nur77 (NR4A1), thereby highlighting its potential as a therapeutic agent [10,11,12,13,14,15,16,17,18,19].

In parallel, microRNA (miRNA) has been increasingly recognized as playing a role in tumor cell proliferation, metastasis, invasiveness, angiogenesis, self-renewal, differentiation, and chemotherapy resistance, which may lead to treatment failure and tumor recurrence [20]. miR-10b was reported to be overexpressed in GBM and shows a pertinent role in GBM progression grading through the downstream targeting of HOXD10/MMP-14/uPAR and the RhoC axis [21,22]. Applying a miR-17-3p, miR-222, and miR-340 combinatorial overexpression efficiently induced cell death and attenuated tumor growth in three GBM subtypes [23].

More recently, attention has turned to the intersection between miRNA regulation and metabolic pathways. For instance, miR-29 was found to regulate the sterol regulatory element-binding protein 1 (SREBP1) [24], encoded by the *SREBF1* gene, in GBM to inhibit SREBP-dependent cholesterol synthesis and uptake, which limits cell survival and tumor formation [25]. Upstream regulation of miRNA is also essential in GBM. miR-29/*SREBF1* forms a negative feedback loop with upstream EGFR signals. It enhances *SCAP-SREBF1* expression, and hence *SREBF1*, as a transcription factor, upregulates the expression of miR-29 [26]. Moreover, the scope of miRNA regulation extends further upstream to non-coding RNAs. For instance, the knockdown of long intergenic non-coding RNA 511 (LINC00511) suppressed miR-15a-5p/*AEBP1* axis in malignant glioma [27]. Additionally, miR-424 and miR-503 can downregulate miR-9, promoting terminal differentiation in acute myeloid leukemia [28].

**Figure 1:**
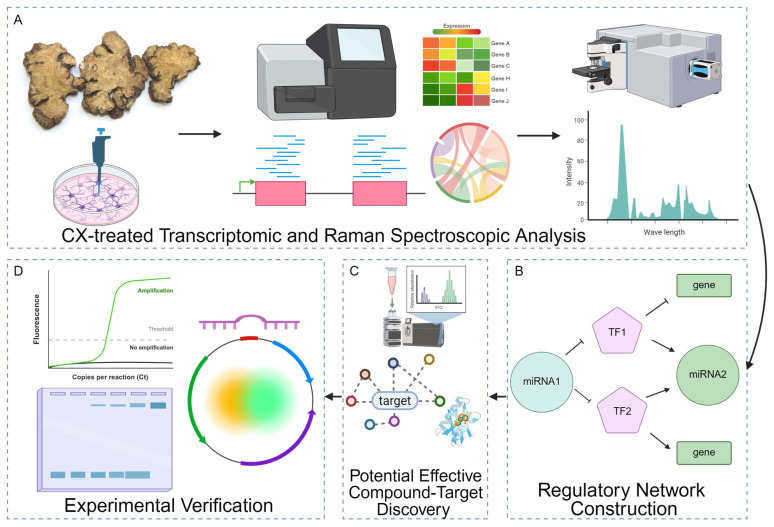
Integrated workflow for dissecting the molecular effects of CX. (**A**) CXG solution-treated transcriptomic and Raman spectroscopic analysis. Cells are treated with the CXG solution, followed by RNA- and small RNA-seq to quantify differential gene expression and Raman microscopy to capture biochemical fingerprints. (**B**) Differentially expressed transcription factors (TFs, pentagons), miRNAs (circles), and target genes (rectangles) are assembled into a mixed TF/miRNA/gene interaction network to prioritize key regulatory motifs. Solid arrows indicate activation and T-bars denote repression. (**C**) Potential effective compounds in the CXG solution were found by UHPLC-MS and network pharmacology in combination with molecular docking. (**D**) Selected molecular axes are validated by quantitative RT-PCR (amplification curve), gel electrophoresis of PCR products, and dual-luciferase reporter assays using plasmid constructs containing miRNA binding sites or TF-responsive promoters.

However, while the critical role of miRNAs in GBM has been established, studies investigating the impact of TCM, particularly CX, on miRNA-mediated regulatory networks remain scarce. Whether CX modulates miRNA expression to influence downstream pathways related to lipid metabolism, collagen deposition, and apoptosis in GBM remains unknown. To address this gap, the present study aims to elucidate the regulatory mechanisms through which CX affects GBM progression, focusing on its influence on miRNA expression and the associated metabolic and apoptotic targets and pathways in combination with transcriptomic analysis, Raman spectroscopy analysis, miRNA network construction, and experimental validation (Figure 1). By uncovering this regulation, we hope to provide new insights into the anti-tumor potential of TCM and highlight novel molecular targets for GBM therapy.

## 2. Results

### 2.1. CXG Solution May Promote Glioblastoma Cell Apoptosis and Suppresses Extracellular Matrix Organization

**Figure 2:**
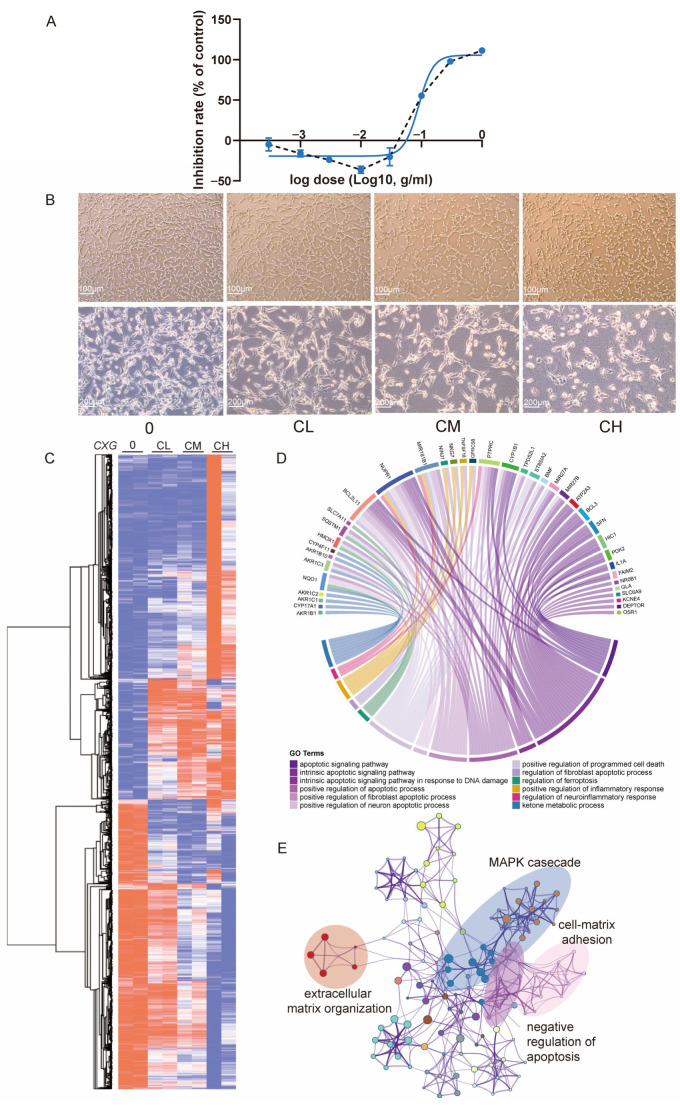
CXG induces cell death and transcriptional reprogramming in U87MG cells. (**A**) Dose–response curve of U87MG cell viability after CXG treatment for 24 h measured by CCK-8 assay (mean ± SD, n = 6). Blue curve: non-linear regression of inhibition rate vs. log10(dose); black dashed line: line graph connecting the data points. (**B**) Representative phase-contrast images showing cell morphology under control (0), low (CL), medium (CM), and high (CH) concentrations of CXG. Scale bars: 100 μm (upper panels) and 200 μm (lower panels). (**C**) Heatmap and hierarchical clustering of DEGs in all three concentrations compared to the non-treated control. Colors represent row-wise normalized expression values (row min, row max; blue–white–red scale). (**D**) Enrichment analysis for downregulated genes and the pathways in which they are involved. Apoptosis-related pathways are denoted in purple. (**E**) Enriched pathways of upregulated genes.

To ensure the quality of the CXG solution, The HPLC-UV method quantified major compounds using commercially available standards, including ferulic acid, senkyunolide A, senkyunolide I, senkyunolide H, and ligustilide. In contrast, UHPLC-MS profiling revealed additional metabolites such as bayogenin, pipecolic acid derivatives, nicotinic acid, and cucurbitacin IIb (Table 1 and Figure S1), which is in concordance with previous studies [6,7]. The dose-dependent effect of the CXG solution on the proliferation of the U87MG cell line was tested by a cell viability assay (Figure 2A). CXG exhibited a clear cytotoxic effect on U87MG cells in a dose-dependent manner. Notably, the curve revealed a biphasic response: at lower concentrations, CXG promoted cell viability, whereas at higher concentrations it markedly reduced viability. As a result, 72.0 (IC_50_, CH), 52.1 (IC_30_, CM), and 31.2 mg/mL (IC_10_, CL) of the CXG solution were used at a series of concentrations in the subsequent experiments. Based on the quantification by HPLC-UV absorption, in CXG, the concentration of ferulic acid in the CXG solution is in the range of CL: 80.277–86.047 μM, CM: 133.802–143.42 μM, and CH: 185.345–198.667 μM with the content of other components shown in Table 2. Among these, ferulic acid and senkyunolide I were the most abundant, whereas ligustilide and levistilide A were present only at trace levels. The morphology of U87MG cells is shown in Figure 2B. With increasing CXG solution concentration, cell density decreased. Cells began to exhibit reduced cell–cell contact and display retraction, resulting in a more rounded morphology.

With the treatment of the CXG solution in a concentration gradient, there were 2168 upregulated and 1842 downregulated differentially expressed genes among CXG solution-treated samples compared with non-treated control (Figure 2C and  Figure S2A,B). Genes differentially expressed under two concentrations (594 upregulated and 802 downregulated) were collected for enrichment analysis (Figure S2C). Major pathways for upregulated genes are apoptosis-related pathways (Figure 2D), with the most related gene being *BCL2L11*. The CXG solution also upregulates pathways related to ketone metabolism and the regulation of ferroptosis. Meanwhile, pathways associated with the negative regulation of apoptosis were downregulated. Other downregulated pathways included oncogenic MAPK cascades and tumor metastasis pathways, such as cell–matrix adhesion and extracellular matrix (ECM) organization (Figure 2E). Survival analysis revealed that lower expression of genes involved in ECM organization, such as *PLAUR*, *TGM2*, *ADAMTSL1*, *FN1*, and *PXN*, is associated with improved overall survival (Figure S3). Notably, *TGM2* and *HMGA2* are also genes involved in lipid metabolism. These results demonstrate the efficacy of the CXG solution in inhibiting extracellular matrix organization and inducing tumor cell apoptosis in the glioblastoma cell line. Previous studies have reported that collagen deposition and ECM remodeling enhance GBM invasion, therapy resistance, and correlate with poor prognosis [29,30]. Lipid metabolism reprogramming, particularly through SREBF1 [26,31], has also been recognized as a hallmark of GBM progression [25]. Our results are consistent with these observations.

### 2.2. The CXG Solution Effectively Downregulates the Collagen and Lipid Synthesis in Glioblastoma Cells

**Figure 3:**
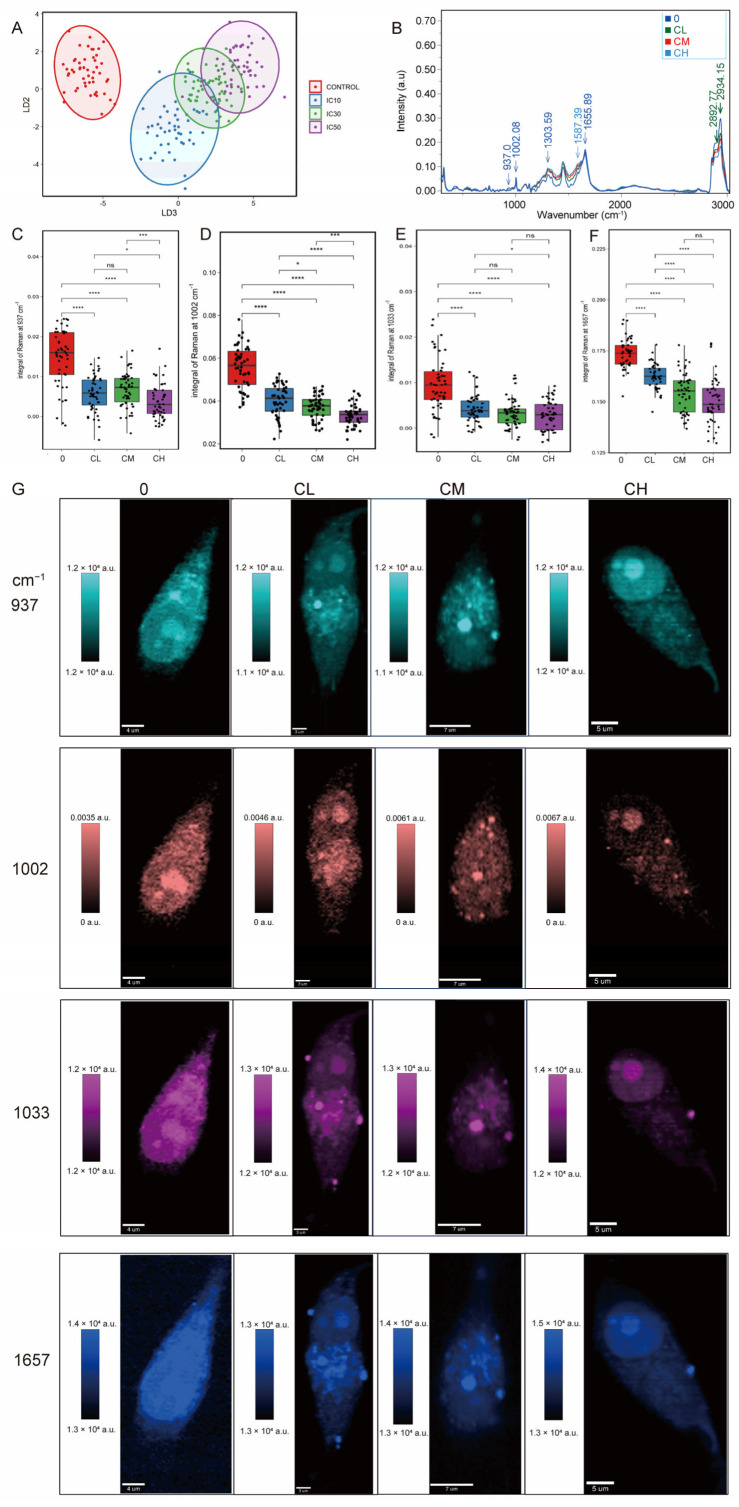
Raman spectroscopic analysis reveals a dose-dependent reduction in collagen synthesis in glioblastoma cells. (**A**) Linear discriminant analysis (LDA) of Raman spectroscopic analysis. (**B**) Mean Raman spectra of different treatment groups. (**C**–**F**) Quantitative comparison of Raman peak (937, 1002, 1033, and 1657 cm^−1^) intensities. Statistical significance was determined by one-way ANOVA. ns, not significant; * *p* < 0.05; *** *p* < 0.001; **** *p* < 0.0001, n = 50 cells. (**G**) Raman mapping of individual cells at selected vibrational frequencies.

The linear discriminant analysis (LDA) plot demonstrated distinct clustering of Raman spectra from glioblastoma cells treated with increasing CXG solution concentrations (CL, CM, CH) compared with the non-treated control. Each point represents an individual cell spectrum, and ellipses indicate each group’s 95% confidence interval (Figure 3A). The progressive separation of clusters indicated dose-dependent biochemical alterations induced by the treatment. Raman spectroscopy revealed that glioblastoma cells treated with increasing drug concentrations exhibited a progressive decrease in the intensities of key Raman peaks (937, 1002, 1033, and 1657 cm^−1^, Figure 3B) corresponding to significant alterations in collagen composition [32]. Single peak integral intensity was quantified and compared in a boxplot (Figure 3C–F), visualizing the signal intensity as Figure 3G. The intensity of the bond vibration under different wavelengths in each condition was detected in a randomly selected cell. Notably, Raman imaging demonstrated a reduction in cytoplasmic projections and a redistribution of intracellular components, suggesting impaired structural integrity and metabolic remodeling under treatment stress (Figure S4).

**Figure 4:**
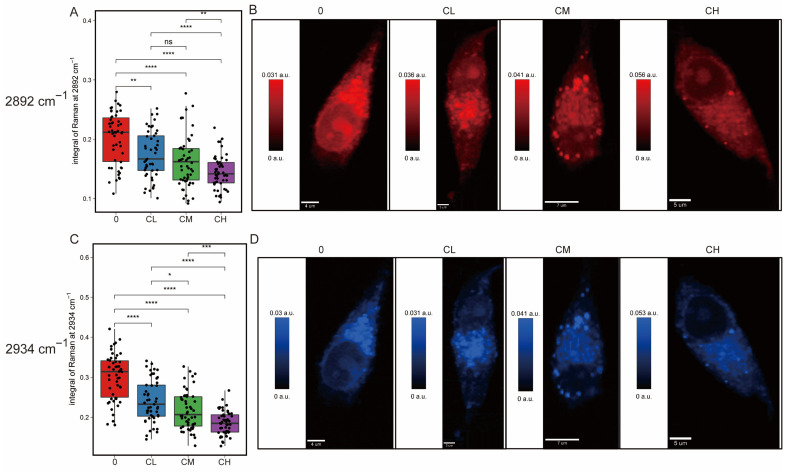
Raman mapping of lipid-associated CH-stretching vibrations in glioblastoma cells under increasing drug treatment. (**A**) Boxplot of integral intensity at 2892 cm^−1^ and (**B**) corresponding Raman map. (**C**) Boxplot of integral intensity at 2934 cm^−1^ and (**D**) corresponding map. Statistical significance was determined by one-way ANOVA. ns, not significant; * *p* < 0.05; ** *p* < 0.01; *** *p* < 0.001; **** *p* < 0.0001, n = 50 cells.

Raman intensity maps of individual cells also highlighted alterations in lipid metabolism and spatial redistribution. Peaks corresponding to CH_2_ symmetric stretching vibrations, characteristic markers of lipid alkyl chains (Figure 4A,B) at 2892 cm^−1^, and CH fatty acid chains (Figure 4C,D) at 2934 cm^−1^ showed a dose-dependent reduction from control (0) to low (CL), medium (CM), and high (CH) drug concentration groups. Results suggested a gradual decrease in saturated lipid content with increasing CXG solution concentration.

Similar phenotypic suppression of ECM remodeling and lipid metabolism has been linked to reduced aggressiveness and increased therapy sensitization in GBM models [33,34]. Taken together, these findings confirm that CXG solution treatment leads to significant alterations in cellular collagen deposition and lipid composition and spatial distribution, supporting the notion that its anti-tumor effect is mediated, at least in part, through the inhibition of collagen deposition and lipid metabolism.

### 2.3. The CXG Solution May Promote Pro-Apoptotic Effects and Reduce Lipid Metabolism Through hsa-miR-10a-5p and hsa-miR-29a-3p

**Figure 5:**
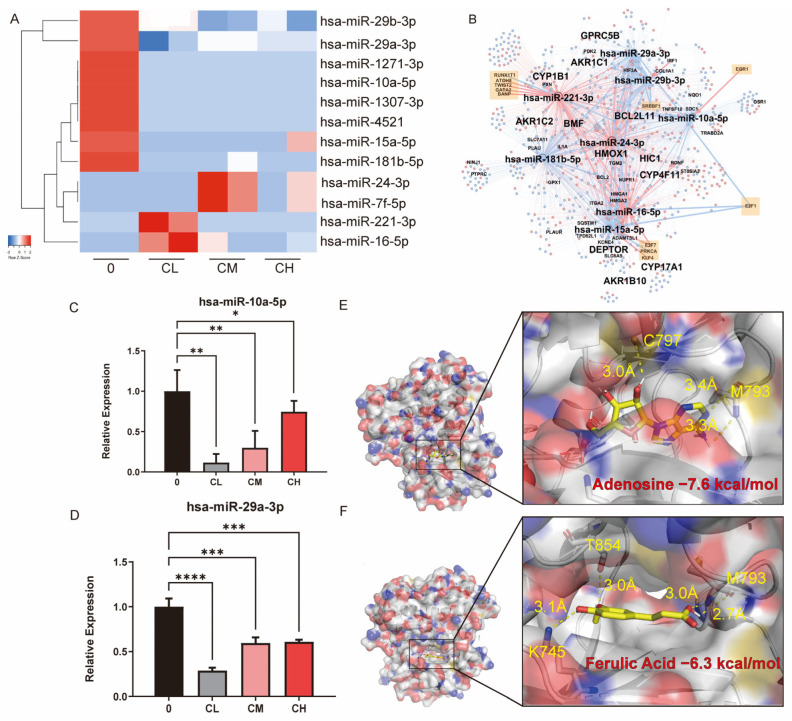
The CXG solution induce cell death and alterations in lipid metabolism in glioblastoma cells via miRNAs. (**A**) Heatmap of mature miRNA expression in control (0), IC_10_ (CL), IC_30_ (CM), and IC_50_ (CH). (**B**) TF-miRNA-target interaction network. Red color nodes represent upregulated genes in CXG solution-treated conditions, while blue nodes represent downregulated genes. Orange squares denote transcription factors. (**C**,**D**) qPCR result of hsa-miR-10a-5p and hsa-miR-29a-3p. (**E**,**F**) molecular docking results and the demonstration of binding pockets of adenosine and ferulic acid to EGFR. Statistical significance was determined by one-way ANOVA. * *p* < 0.05; ** *p* < 0.01; *** *p* < 0.001; **** *p* < 0.0001.

Small RNA-seq was performed to explore how CX regulates miRNAs. The results showed that the CXG solution downregulated eight miRNAs and upregulated four miRNAs (Figure 5A). The TF-miRNA-target regulatory network is shown in Figure 5B. Within the network there are 12 pairs of TF-miRNA regulations and 2172 pairs of miRNA-target regulations. This network indicates that a feed-forward loop regulation exists between hsa-miR-10a-5p and hsa-miR-29a-3p, mediated through targeting *E2F1* and *SREBF1*. The expressions of hsa-miR-10a-5p and hsa-miR-29a-3p were verified by qPCR. The CXG solution significantly downregulated their expression, which was consistent with the sequencing data (Figure 5C,D). To place these findings in context, we next compared them with the previously reported roles of miR-10a-5p and miR-29a-3p in glioblastoma. It has been reported that miR-10a-5p is frequently overexpressed in GBM and promotes proliferation and migration [35,36], whereas miR-29a-3p acts as a tumor suppressor and inhibits lipogenesis [37]. Our results, therefore, align with these previous findings, supporting the role of CX in modulating miRNA-driven pro-apoptotic effects and metabolic reprogramming. Among them, *BCL2L11*, *E2F1*, and *SREBF1* were confirmed to be significantly regulated by the CXG solution. The downregulation of hsa-miR-10a-5p was accompanied by increased BCL2L11, while *E2F1* and *SREBF1* were suppressed, and the regulation of hsa-miR-29a-3p was subsequently verified. Because miRNA-profiling pointed to an *E2F1-centered* transcriptional program controlling the downregulated lipid-metabolic miRNAs, we next searched the network for proteins that (i) are directly modulated by the detected compounds, (ii) lie immediately upstream of, and (iii) negatively modulate E2F1. For each compound, we queried compound–target interaction (CTI) pairs from ChEMBL (v35), BindingDB (July 2025 release), PubChem BioAssay, and a literature-sourced CTI dataset [38]. After de-duplication, we obtained 406 unique human CTI pairs (Table S1). CTIs and E2F1-interacting genes data were assembled into a bipartite network (Figure S5). Hub analysis highlighted nicotinic acid (k = 17) and ferulic acid (k = 12) as the most multi-target phytochemicals. At the same time, MAPK1, EGFR, ESR1, and PPARG were the four highest-degree protein nodes (k ≥ 6), indicating that the CXG solution may converge on classic growth-factor and nuclear-receptor signaling axes. EGFR emerged as the sole node that satisfied all criteria, which is inhibited by adenosine and ferulic acid according to ChEMBL bioassay data. The inhibition of EGFR was reported to inhibit the activity of E2F1 in cancers [39]. Molecular docking results show that the Vina score of binding to EGFR is −7.3 kcal/mol for adenosine and −6.3 kcal/mol for ferulic acid (Figure 5E,F).

**Figure 6:**
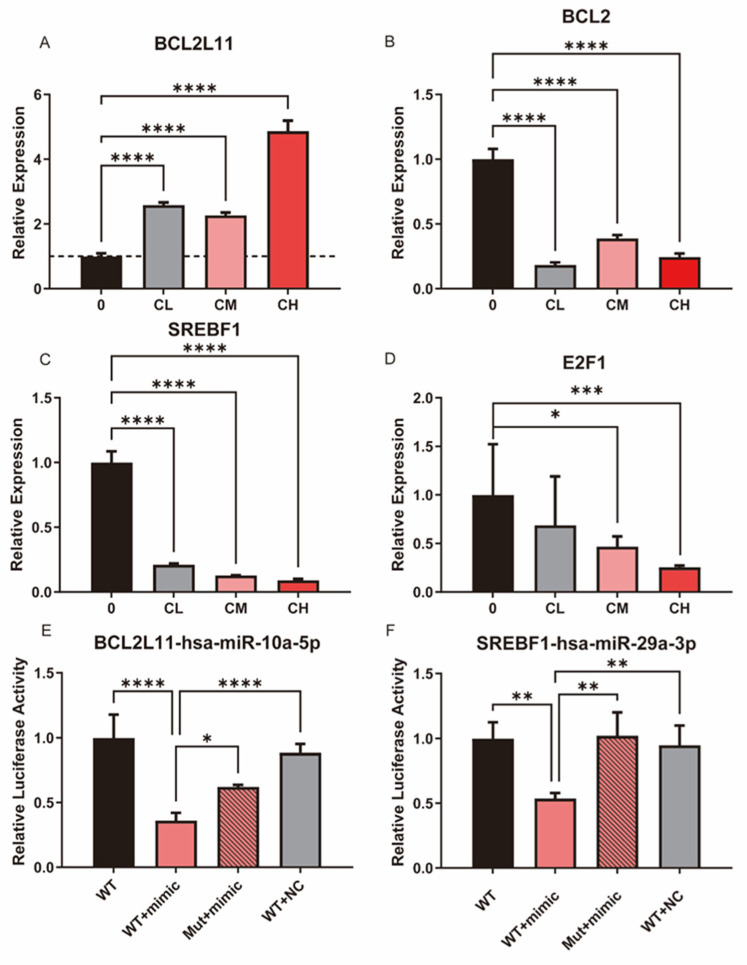
The CXG solution may promote apoptosis and suppress lipid metabolism through a miRNA-involved regulatory network. (**A**–**D**) qPCR results of BCL2L11, BCL2, SREBF1, and E2F1 following CXG solution treatment. The dashed line indicates a relative expression level of 1 in the qPCR results. (**E**,**F**) Dual-luciferase reporter assay validated miRNA-mRNA interactions of hsa-miR-10a-5p/BCL2L11 and hsa-miR-29a-3p/SREBF1. Statistical significance was determined by one-way ANOVA. * *p* < 0.05; ** *p* < 0.01; *** *p* < 0.001; **** *p* < 0.0001.

The expression levels of key apoptosis- and lipid metabolism-related molecules in U87MG glioblastoma cells following treatment with the CXG solution were evaluated. qPCR results indicated elevated levels of pro-apoptotic genes (i.e., *BCL2L11*) alongside the decreased expression of anti-apoptotic markers (i.e., *BCL2*) with increasing CXG solution dosage, consistent with the activation of cell death-associated transcriptional programs (Figure 6A,B). Figure 6C,D highlight the suppression of critical lipid metabolism regulators, such as *SREBF1* and *E2F1*, suggesting that the CXG solution impairs fatty acid or cholesterol biosynthesis. Notably, *BCL2* and *SREBF1* mRNA levels decreased already at the lowest CXG concentration (CL), where cell viability was largely unaffected, indicating a lack of strict CXG dose-dependence. A dual-luciferase reporter assay validated the miRNA-mRNA interactions of hsa-miR-10a-5p/*BCL2L11*, and hsa-miR-29a-3p/*SREBF1*. There was a significant reduction in luciferase activity when the wild-type 3′-UTR was present with the miRNA mimic introduced, indicating direct binding to the predicted site (Figure 6E,F). These data provide supporting evidence that hsa-miR-10a-5p directly regulates *BCL2L11* as a pro-apoptotic mediator, which is in concordance with previous studies [40,41], highlighting the regulatory mechanism of the CXG solution through the miRNA regulatory network.

**Figure 7:**
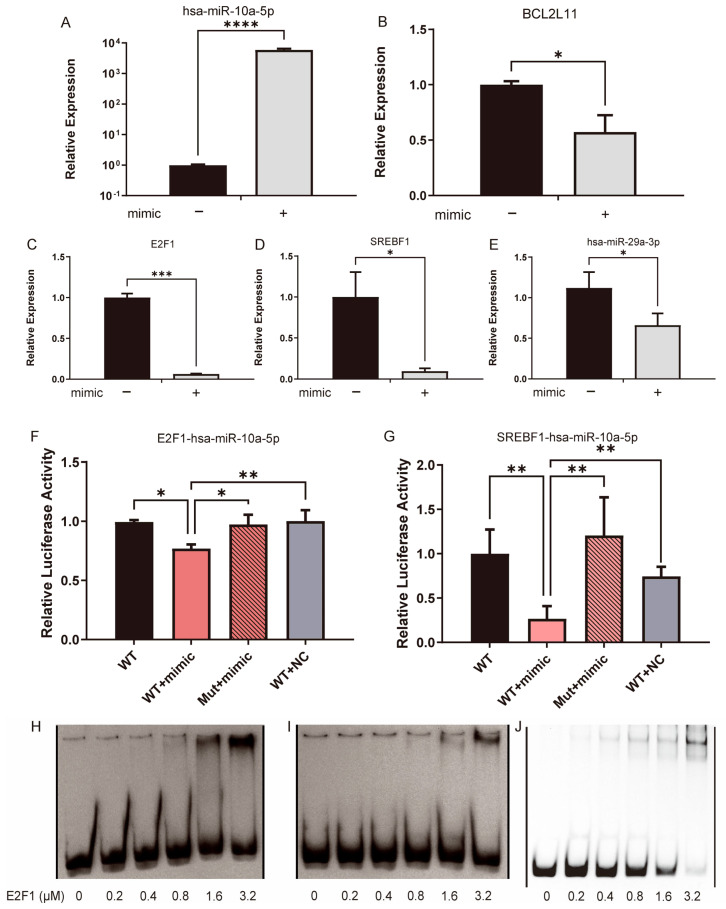
The **C**XG solution and hsa-miR-10a-5p downregulated hsa-miR-29a-3p by the coherent feed-forward loop. (**A**–**E**) qPCR result compared gene expression with or without the transfection of hsa-miR-10a-5p. (**F**,**G**) Dual-luciferase reporter assay verifies MTI hsa-miR-10a-5p/E2F1 and hsa-miR-10a-5p/SREBF1. Statistical significance was determined by one-way ANOVA. * *p* < 0.05; ** *p* < 0.01; *** *p* < 0.001; **** *p* < 0.0001. (**H**) An EMSA shift verified the interaction between E2F1 and the hsa-miR-29a promoter region. (**I**) The shift is not apparent when the promoter region is mutated. (**H**,**I**) are derived from the same gel. One irrelevant lane between the WT probe (**H**) and mutant probe (**I**), and one empty irrelevant lane at the left and right, respectively, were removed. A thin black line indicates the splice. (**J**) An EMSA shift in positive control. One irrelevant empty lane on the left and eight irrelevant empty lanes on the right were removed and are indicated by a thin black line.

As the CXG solution downregulated hsa-miR-10a-5p, the regulation between hsa-miR-10a-5p and *BCL2L11* was further verified by the restoration of hsa-miR-10a-5p. qPCR results showed that when hsa-miR-10a-5p was restored (Figure 7A), expression of *BCL2L11* was downregulated in the U87MG cell line (Figure 7B). Notably, *E2F1*, *SREBF1*, and hsa-miR-29a-3p expressions were also downregulated (Figure 7C–E). As associated in the TF-miRNA-target regulatory network, *E2F1* and *SREBF1* are also downstream targets of miR-10a-5p, which, to our knowledge, are validated for the first time by a dual-luciferase reporter assay (Figure 7F,G). By an EMSA, we further assessed the binding of the E2F1 protein as a transcription factor in the promoter region of hsa-miR-29a-3p, as predicted. Figure 7H shows an evident shift in the probe when the protein concentration was larger than 1.6 μM, whereas the mutated probe did not show a shift at 1.6 μM. It was not significant at 3.2 μM (Figure 7H,I). Figure 7J shows a sharp shift gradient as the positive control of the probe was input, which contains a known E2F1-binding region [42]. As the regulation of *SREBP1* by hsa-miR-29a has been previously reported [26], the hypothesis was proposed that the expression of hsa-miR-29a-3p can be suppressed by the CXG solution and hsa-miR-10a-5p through the downregulation of its transcription factors, *SREBF1* and *E2F1*, in a coherent feed-forward loop, thereby orchestrating pro-apoptotic effects and metabolic reprogramming.

## 3. Discussion

**Figure 8:**
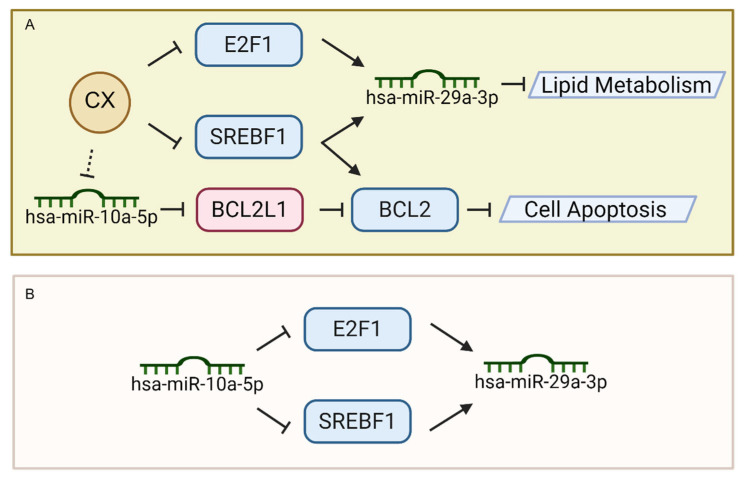
Regulatory mechanism of CX in promoting cell death and suppressing lipid metabolism by feed-forward loop. (**A**) Mechanism of CX in regulating lipid metabolism and cell apoptosis. (**B**) Feed-forward loop of hsa-miR-10a-5p in suppressing hsa-miR-29a-3p by targeting transcription factors.

In this study, we demonstrated that the CXG solution exerts anti-tumor effects on GBM cells, manifested primarily through the potential induction of apoptosis and the suppression of lipid metabolism and ECM organization. Our multi-omics approach, including transcriptomics and small RNA sequencing, and Raman spectroscopy, provided convergent evidence of the CXG solution’s efficacy in targeting GBM via key molecular axes, particularly the hsa-miR-10a-5p/*BCL2L11*/*BCL2* and CX/*SREBF1*/*E2F1*/hsa-miR-29a-3p feed-forward loop. *BCL2L11* encodes BIM, a pro-apoptotic BH3-only protein that directly antagonizes BCL2 and promotes the activation of Bax/Bak, thereby linking our observed transcriptional changes in *BCL2L11* and *BCL2* to the intrinsic apoptotic pathway, while our intention was not to imply the direct transcriptional regulation of *BCL2* by *BCL2L11*, but rather to illustrate a potential regulatory axis through protein–protein interactions suggested by the transcriptomic data. Previous studies have reported on the anti-glioma activities of CX and its constituents, including tetramethylpyrazine, ligustilide, and ferulic acid, which inhibit proliferation, invasion, and sensitize GBM to temozolomide via CXCR4, Rho GTPases, and PI3K/Akt pathways [10,11,12,13,14,15,16,17,18,19]. From a mechanistic viewpoint, this research brought the role of miRNAs in mediating CX’s anti-tumor activities into sharper focus (Figure 8A).

Notably, this feed-forward loop not only underscores the central role of hsa-miR-10a-5p in modulating lipid metabolism and apoptosis-related factors (e.g., *SREBF1*, *E2F1*) [43,44] but also highlights how upstream miRNA regulation can ultimately control the expression of another downstream miRNA (hsa-miR-29a-3p). However, the importance of higher-level regulation, particularly in miRNA-TF-miRNA loops, has often been underappreciated. However, prior studies have extensively investigated miRNA-mRNA interactions. Our findings demonstrate that hsa-miR-10a-5p serves as an upstream regulator in a coherent feed-forward loop (Figure 8B), paving the way for a more nuanced understanding of how multiple layers of non-coding RNAs and transcription factors synergistically govern glioblastoma cell fate.

The CXG solution significantly reduces the expression of ECM-related genes (e.g., *FN1*, *TGM2*, and *PXN*) and collagen synthesis pathways, as suggested by RNA-seq data and Raman spectroscopy. Survival analyses in our cohort further showed that a lower expression of ECM-related genes correlated with prolonged survival, consistent with the literature [45,46]. Our survival analysis further showed that a lower expression of ECM-related genes correlates with prolonged overall survival in GBM, underscoring the potential therapeutic value of inhibiting ECM organization and remodeling. CXG solution treatment thus limits cell proliferation and diminishes invasive capacities by disrupting the ECM.

Another important aspect of this work is the explicit demonstration of the CXG solution-mediated suppression of lipid metabolism. Emerging evidence suggests that lipid-based therapeutic molecules are promising research targets for unraveling novel drugs in GBM [47]. The *SREBF1* gene encodes the nuclear transcription factors SREBP-1a and SREBP-1c, which are released from the endoplasmic reticulum membrane when cellular sterol levels decrease or insulin/carbohydrate signals increase. Once in the nucleus, SREBP-1 binds sterol-response elements and potently upregulates the core enzymes of de novo fatty-acid and triglyceride synthesis, thereby coordinating lipid storage, very-low-density lipoprotein assembly, and adipocyte differentiation [48]. As SREBP plays a key role in GBM sterol lipid regulation, its inhibitor fatostatin has been reported to induce ferroptosis in GBM [49]. Additionally, it was reported that E2F1 promotes the proliferation and metastasis of clear cell renal cell carcinoma by activating SREBP1-dependent fatty acid biosynthesis [50]. Our findings indicate that the CXG solution effectively downregulates key lipid regulatory genes, including *SREBF1*, via an FFL mediated by the hsa-miR-10a-5p/*E2F1* axis. Raman spectrometric mapping corroborated these transcriptomic results, revealing dose-dependent decreases in lipid-associated signals in GBM cells treated with the CXG solution. Our findings that the CXG solution diminishes lipid synthesis genes and lipid-associated signals are therefore consistent with and extend these prior reports.

Despite these promising findings, several limitations warrant consideration. First, the current study was limited to in vitro experiments in a single GBM cell line (U87MG). The intricate heterogeneity of GBM necessitates validation in multiple cell lines, patient-derived primary cell lines, organoids, or animal models before clinical relevance can be firmly established. Second, although we identified multiple bioactive compounds in CX, the specific contribution of each constituent to the observed anti-GBM effects remains to be elucidated. Network pharmacology and molecular docking results indicate that adenosine and ferulic acid bind to EGFR, acting as an upstream suppressor of E2F1 and hsa-miR-10a-5p. In our study, the concentration of the CXG solution was calculated based on the original mass of CX. While the IC_50_ values indeed appear high compared to many purified phytochemicals, quantitative HPLC-UV data (Table 2) revealed that ferulic acid and senkyunolide I are relatively abundant (tens to hundreds of μM), whereas ligustilide and levistilide A are present at trace levels. For instance, ligustilide was detected in CXG at 0–8.6 µM, whereas previous studies have reported that ligustilide inhibits the migration of T98G glioma cells at 5 µM without inducing cell proliferation or apoptosis [51], and exerts cytotoxic effects with an IC_50_ of approximately 20 μg/mL in U251 glioma cells [19]. Similarly, ferulic acid was quantified in our CXG solution at 80–199 μM, which is higher than the reported concentration that induces apoptosis in GBM cells (~36 μM [18]) yet far below the reported IC_50_ concentration of 4.706 mM for U87MG cells [52]. These comparisons suggest that the nominal concentrations used in our study are within a biologically relevant range for its key constituents, once their relative abundance in the crude extract is considered. The results highlight that only a small fraction of CXG contributes to its anti-GBM activity. Interestingly, within the CXG mixture in concentration of IC_50_, the measured concentrations of key constituents such as ferulic acid and ligustilide were below or close to the IC_50_ values reported for these compounds when tested individually in glioma cells. This observation suggests that CXG exerts its bioactivity through additive or synergistic interactions among multiple components, allowing the overall preparation to achieve cytotoxic effects at concentrations where the individual compounds would otherwise be sub-effective. Such synergy aligns with the TCM principle that therapeutic efficacy results from the coordinated modulation of multiple pathways rather than from single-target inhibition.

Third, while our rationale in this work was to evaluate CXG in its clinically used composite form, consistent with the Traditional Chinese medicine (TCM) principle of considering the overall pharmacological effect of the preparation rather than isolated compounds. Consequently, the concentrations in our study were expressed as raw herb equivalents of Chuanxiong rhizome (mg/mL), which appear higher than those reported for lyophilized ethanol/water extracts, extracts fractions, and hydrolyzed extracted powder. For example, Hu et al. reported that the IC_50_ values of CX fractions against HepG2 and SMMC7721 cells, as determined by an in vitro MTT assay, were approximately 100 μg/mL for CX polysaccharide fractions in HepG2 and SMMC7721 cells, corresponding to 0.5–1 mg of crude herb equivalent [53]. In the same study, IC_50_ values of CX lyophilized polysaccharide fractions in other cancer cell lines, such as HCT-116 and A549, were reported to be higher, reaching ~500–1000 μg/mL (2.5–5 mg/mL CX equivalent), indicating that the effective concentrations of CX fractions vary by cancer type [53]. These complexities underscored the need for fractionation and more targeted studies to pinpoint the most potent chemical entities.

Fourth, pharmacokinetic constraints must be considered. While we have not yet measured the plasma levels of ferulic acid after the administration of CXG in animal models or human subjects, published pharmacokinetic studies of ferulic acid or its precursors/metabolites showed that in vivo plasma concentrations are many orders of magnitude lower than the cell culture concentrations used. Ferulic acid was rapidly absorbed following oral administration with a mean time to peak plasma concentration (Tmax) of 0.03 h. The corresponding maximum plasma concentration (Cmax) and the area under the concentration–time curve (AUC) were 8.175 μg/mL (42.1 ± 5.2 μM) and 2.595 μg·h /mL, respectively [54]. Further in vivo pharmacokinetics and formulation strategies should be addressed to enhance bioavailability and blood–brain barrier penetration in future work. To better reflect the pharmacologically relevant conditions, future studies could evaluate combinations of major CXG constituents at their experimentally determined plasma concentrations or in ratios corresponding to their abundance in CXG extracts. This strategy would enable the simulation of the integrated, multi-component pharmacology of TCM and help bridge in vitro efficacy with in vivo exposure. Moreover, systematic testing of these physiologically relevant combinations could clarify whether the enhanced potency of CXG arises from compound synergy, complementary target engagement, or improved cellular uptake, providing a framework for PK-PD modeling and translational optimization. In addition, we acknowledge the absence of non-cancerous proliferating cell controls, which would strengthen conclusions regarding selectivity. Previous work reported that the in vitro IC_50_ in mouse embryonic fibroblasts (3T3) of *Chuanxiong Rhizoma* decoction was 9.39 mg/mL, and the IC_50_ for mouse embryonic stem cell D3 was 18.78 mg/mL [55]. Although such experiments were not performed here, we propose to address this in future work, particularly by testing hematopoietic or neural progenitor cell models.

Furthermore, the observed effects may represent a biphasic, concentration-dependent response (hormesis). Low concentrations promote metabolic activity or proliferation, while higher concentrations induce cell death. This dual behavior is consistent with the traditional TCM concept of “huo xue xing qi” (“invigorating blood circulation and promoting energy flow”), which emphasizes regulatory rather than exclusively inhibitory functions. In addition, as shown in Figure 6B,C, *BCL2* and *SREBF1* expression were already significantly reduced at the lowest CXG concentration (CL), while cell viability was largely unaffected. This apparent lack of dose-response, together with the biphasic effect, is likely attributable to the complex and potentially opposing actions of multiple components within CXG. Accordingly, the present manuscript focuses primarily on elucidating the cytotoxic mechanisms of CXG at high concentrations, while the cytoprotective or growth-promoting effects observed at lower concentrations represent an interesting and valuable direction for future investigation. We also recognize that elevated concentrations raise concerns regarding nonspecific or nonselective effects. In the case of CXG, this may also reflect its complex, multi-component nature, where different constituents can exert opposing activities (e.g., cytoprotective versus proapoptotic) depending on the concentration. Thus, what appears as nonspecific cytotoxicity may instead represent the overlapping effects of multiple active molecules, rather than technical assay interference, highlighting that the complex pharmacology of TCM formulations inherently involves multi-target interactions, and systematic investigations remain necessary.

Finally, while our data suggest a regulatory role of miRNAs in lipid metabolism and apoptosis, additional apoptosis-specific assays, protein-level analyses, and in vivo functional assays are needed to further strengthen mechanistic interpretation. Accordingly, the proposed hsa-miR-10a-5p/BCL2L11/BCL2 and CX/SREBF1/E2F1/hsa-miR-29a-3p axes should be regarded as hypothesis-generating models, and we explicitly recognize that protein-level validation will be essential in future studies to establish causality. From a translational standpoint, CX compounds may complement existing GBM therapies, particularly by modulating lipid biosynthesis and ECM remodeling pathways that are increasingly recognized as therapeutic vulnerabilities. By fine-tuning miRNA networks and transcriptional regulators, CX may offer a valuable complementary approach to standard-of-care treatments, such as temozolomide or radiotherapy. Future research should address the pharmacokinetics, blood–brain barrier permeability, and synergistic potential of CX-derived compounds in combination with current GBM therapeutics.

## 4. Materials and Methods

### 4.1. HPLC-UV and LC-MS/MS Analysis of the CXG Solution

Chuanxiong dispensing granules (CXG) were purchased from E-Fang Pharmaceutical Co., Ltd. (Foshan, China). According to the company’s statement and national guidelines, CXG was produced from authenticated crude CX. The manufacturing process involves (i) water decoction under controlled conditions, (ii) extraction of essential oils by steam distillation, (iii) recombination of volatile oil with the aqueous extract, (iv) concentration and spray-drying to obtain dry extract powder, and (v) dry granulation to yield instant granules. This procedure follows the Chinese Pharmacopoeia requirements for dispensing granules and ensures chemical equivalence to the corresponding traditional decoction. According to the manufacturer’s equivalence, 0.217 g CXG corresponds to 1 g of dried rhizome of *Ligusticum chuanxiong* (as per the Chinese Pharmacopoeia standard). CXG was dissolved in serum-free DMEM medium to reach a final concentration 0.01 g/mL of CXG (around 46 mg/mL of crude CX). The suspension was incubated at 37 °C with gentle shaking and centrifuged at 12,000 rpm for 5 min. The supernatant was collected and designated as the CXG solution, which was used for HPLC-UV and LC**-**MS/MS and all in vitro experiments. The system for HPLC-UV absorption consisted of a Shimadzu LC-20AT equipped with a UV detector (SPD-20A, Shimadzu, Kyoto, Japan). Separation was carried out on a C18-A column (4.6 × 250 mm, 5 µm, Cat# DC952505-0, Agela Technologies, Tianjin, China) maintained at 30 °C. The mobile phase consisted of solvent A (water with 0.1% phosphoric acid, Cat# J2210679, Aladdin, Shanghai, China) and solvent B (acetonitrile, 99.90%, Cat# CAEQ-4-003306-4000, CNW, Shanghai, China), with the following gradient program: 0.00–1.00 min, 8% B; 1.00–7.00 min, 8–12% B; 7.00–12.00 min, 12–18% B; 12.00–20.00 min, 18–30% B; 20.00–24.00 min, 30% B; 24.00–32.00 min, 30–45% B; 32.00–40.00 min, 45–60% B; 40.00–47.00 min, 60–75% B; 47.00–50.00 min, 75–80% B; and 50.00–60.00 min, 80% B, followed by re-equilibration at 8% B for 8 min (60–68 min). The flow rate was set at 1.0 mL/min, and the injection volume was 10 μL. Detection wavelengths were selected according to the maximum absorbance of each compound, including 320 nm for ferulic acid, 325 nm for ligustilide, 277 nm for senkyunolide I, 278 nm for senkyunolide A, 276 nm for senkyunolide H, and 275 nm for levistilide A. Quantification was performed using external calibration curves constructed with authentic standards (ferulic acid (Cat# DST-DY0009-0020, Desite, Chengdu, China. The same supplier was used for all other standard compounds.), senkyunolide A (Cat# DST-DY0008), senkyunolide I (Cat# DST-DY0009-0020), senkyunolide H (Cat# DST-DY0182-0020), and ligustilide (Cat# DST-DH0007), and levistilide A (Cat# DST-DO0001-0020)). Each calibration curve exhibited good linearity (R^2^ > 0.999) within the tested concentration range. The compound contents in the samples were calculated from the corresponding calibration equations and expressed as mg/g of extract with 95% confidence intervals (95% CI).

LC-MS and MS/MS analyses of the CXG solution were performed using a high-resolution tandem mass spectrometer Q-Exactive connected to a Vanquish Flex UHPLC system (Thermo Fisher Scientific, Bremen, Germany). An ACQUITY UPLC T3 column (100 mm × 2.1 mm, 1.7 µm, Waters, Milford, MA, USA) was used for the reversed-phase separation. The flow rate for UHPLC was 0.3 mL/min, and the mobile phase consisted of solvent A (water, 0.1% formic acid) and solvent B (Acetonitrile, 0.1% formic acid). Gradient elution conditions were set as follows: 0–0.8 min, 2% B; 0.8–2.8 min, 2–70% B; 2.8–5.6 min, 70–90% B; 5.6–6.4 min, 90–100% B; 6.4–8 min, 100% B; 8–8.1 min, 100–2% B; and 8.1–10 min, 2%B. The Q-Exactive was operated in both positive and negative ion modes.

### 4.2. Cell Culture and Cell Viability Assay

A human glioblastoma-like astrocytoma U87MG cell line (Cat# CC1703, Cellcook, Guangzhou, China) was purchased and cultured in high-glucose Dulbecco’s Modified Eagle Medium (Cat# 11965092, Gibco, Grand Island, NY, USA) supplemented with 10% fetal bovine serum (FBS, Cat# A5256701, Gibco) and 1% penicillin/streptomycin (Cat# 15140122, Gibco) at 37 °C with 5% CO_2_. The cell line was originally established from a female patient with glioblastoma and is widely used as a representative in vitro model for GBM research. U87MG cells display high proliferative and invasive capacities, closely mimicking the aggressive nature of GBM. The anti-tumor effects of the CXG solution were evaluated using a cell viability assay. The U87MG cell line was seeded at a density of 1.0 × 10^4^ cells/well in 96-well plates. After 24 h incubation, the suspension was replaced with fresh medium (control group, 0 μg/mL) or drug solution (treatment groups) at a series of doses of herbal decoction pieces (0.0003, 0.001, 0.003, 0.01, 0.03, 0.1, 0.3, and 1 g/mL). After another 24 h of incubation, the suspension was replaced with 100 μL/well CCK-8 (Cat# C0038, Beyotime, Shanghai, China) solution at a ratio of 1:9 and incubated for 1 h at 37 °C. The microplate reader (BioTek Epoch 2, BioTek, Winooski, VT, USA) measured the absorbance at a wavelength of 450 nm. For the subsequent sequencing experiments in this study, the CXG solution was used at a series of concentrations according to the cell viability inhibition rate: 72.0 mg/mL (IC_50_, CH), 52.1 mg/mL (IC_30_, CM), and 31.2 mg/mL (IC_10_, CL) (Table 3). IC50 (half-maximal inhibitory concentration) was used to measure the efficacy of the CXG solution’s efficacy, which describes the amount of CX needed to inhibit the growth of cancer cells by half.

### 4.3. Raman Spectroscopy

Raman spectroscopy was performed on U87MG glioblastoma cells treated with CXG at different concentrations (0, CL, CM, and CH). A micro-Raman Spectrometer (Deuterium Peak Medical Instrument Co., Ltd., Shanghai, China, Model: CD100) was applied with a 532 nm laser, grating: 600 g/mm, spectral range: −203 to 3724 cm^−1^, laser power: 20 mW, integration time: 3 s, 63× water immersion objective, and spot size: approximately 300 nm. Before data collection, the instrument was calibrated using a silicon wafer, adjusting the silicon signal to 520.7 cm^−1^.

### 4.4. Transcriptomic Sequencing and Data Analysis

When cells reached 70–80% confluency, total RNA from U87MG was harvested using the TRIzol agent (Cat# 15596026, Invitrogen, Carlsbad, CA, USA) and assessed for integrity using a 2100 Agilent Bioanalyzer (Agilent, Santa Clara, CA, USA) before library preparation. A spectrophotometer (Implen NanoPhotometer^®^ N60/N50, Munich, Germany) was used to measure the concentration of RNA. The sequencing was performed as paired-end reads of 100 bp (PE100) on the BGISEQ-500 platform for subsequent data analysis. The sequencing depth was approximately 6 Gb.

Small RNA was first extracted using a total RNA extraction kit (Cat# DP761, TIANGEN, Beijing, China). Libraries were constructed using the MGIEasy Small RNA Library Prep Kit (MGI, Shenzhen, China) following the manufacturer’s instructions. Briefly, 18–30 nt small RNAs were size-selected by PAGE gel purification and ligated to 5′ and 3′ adapters. Reverse transcription was performed to generate cDNA, followed by PCR amplification and purification. The resulting libraries were quantified, pooled, and sequenced on the BGISEQ-500 platform (BGI, Shenzhen, China) to generate single-end 50 bp reads. The sequencing depth was around 10 M.

RNA-seq data processing: Low-quality reads and adaptor sequences were filtered and trimmed using Trim Galore! v0.6.8. After removing ribosomal RNA using SortMeRNA [56], the cleaned reads were aligned to the reference genome hg38 using bowtie2. An mRNA count matrix was generated to perform differential expression analysis. The matrix was normalized by count per million (CPM) within samples to reduce the inter-sample difference. Differentiation analysis was performed by the R (version 4.2) package DESeq2 (version 3.15) [24] with |log2FC| ≥ 1. The *p*-value was calculated and adjusted with the Benjamini–Hochberg algorithm, selecting differentially expressed genes (DEGs) with FDR ≤ 0.1.

Small RNA-seq data processing: the low-quality reads and adaptor sequences in raw reads were filtered and trimmed using Trim Galore!. Next, the cleaned reads were aligned against miRbase [57], mature miRNA, and hairpin using Bowtie. The expression matrix of mature miRNA expression was obtained, and the low-expression reads with a summary CPM less than 10 in all samples were removed. Differentially expressed miRNA (DEmiRs) was performed using DESeq2 with |log2FC| ≥ 1. The *p*-value was calculated using the default method and adjusted with the Benjamini–Hochberg algorithm, selecting DEmiRs with an FDR ≤ 0.1.

### 4.5. miRNA-Target Prediction

To further analyze the miRNA and mRNA profiles in our study, we explored the relationship between the screened DEGs and DEmiRs through miRTarBase [58] and the prediction programs TargetScan [59], miRDB [60], miRWalk [61], and miRTarBase [58].

### 4.6. Transcription Factors Prediction

Human transcription factors were retrieved from the TRANSFAC Professional database [62] and intersected with DEGs and the predicted miRNA targets. miRNA transcription factors were obtained from TransmiR2.0 [63].

### 4.7. Compound–Target Interaction Network Construction and Molecular Docking

Pairs of compound–target interactions were collected from ChEMBL (v35) [64], BindingDB (July 2025 release) [65], PubChem BioAssay [66], and a literature-sourced CTI dataset [38]. The direct targets of the compounds were then subjected to a protein–protein interaction network construction by STRING v12.0 [67]. Molecular docking analysis at the canonical binding pocket was conducted by AutoDockTools 1.5.6 (ADT) [68], specifically AutoDock Vina. The protein structure of EGFR (PDB: 1XKK, resolution 2.4 Å) was retrieved from the Protein Data Bank (RCSB PDB, www.rcsb.org/). The 3D coordinates of anticipated active compounds were retrieved from the PubChem database (Pubchem CID: adenosine: 60961; ferulic acid: 445858).

### 4.8. Regulatory Network Construction

A TF-miRNA-target regulatory network was constructed by combining transcription factors in DEGs and DEmiRs. Gephi v10 [69] was used for network visualization.

### 4.9. Gene Functional Enrichment Analysis

Gene Ontology (GO) analysis on biological processes was performed by Metascape [70] and Metacore (Clarivate, Philadelphia, PA, USA) to observe the enriched biological pathways of DEGs between the control and CXG solution to identify the pathways regulated. FDR ≤ 0.05 was adopted.

### 4.10. Survival Analysis

GEPIA2 [33] is an interactive web server that contains 9736 tumor samples and 8587 normal samples from the TCGA and GTEx projects, providing differential expression analysis between tumor and normal tissues. Survival analysis of identified DEGs in 82 GBM tumors was obtained with 50 percent quantile and log-rank *p*-value < 0.01 as the cutoff values.

### 4.11. Quantitative Reverse Transcriptase-PCR (qPCR)

Total RNAs of U87MG were harvested when cells reached 70–80% confluency by TRIzol agent (Cat# 15596026, Invitrogen). Total RNA was extracted per the manufacturer’s instructions using the Direct-zol^TM^ RNA miniprep kit (Cat# R2052, Zymo Research, Irvine, CA, USA). A spectrophotometer (Implen NanoPhotometer^®^ N60/N50, Munich, Germany) was used to measure the concentration of RNA. Next, first-strand cDNA was generated with 1 μg total RNA using Super Script III Reverse Transcriptase (Cat# 12574026, Invitrogen) in a 20 μL PCR system. A volume of 2 μL of the product was subjected to amplification using Applied Biosystems QuantStudioTM 6 with PowerUp SYBR Green Master Mix (Cat# A25742, Applied Biosystems™, Waltham, MA, USA). The primers used are listed in Table 4.

### 4.12. Dual-Luciferase Reporter Assay

A dual-luciferase reporter assay was performed to validate the miRNA-mRNA interaction. miRWalk [71] was used to predict the binding side. HEK-293 cells (Cat# CC4002, cellcook) in 24-well plates were transfected with 0.1 μM miRNA mimics and 0.5 μg pmiRGLO using Lipofectamine 2000 (Cat# 11668019, Invitrogen). HEK-293 cells were lysed after 24 h, and activity was determined using a dual-luciferase assay kit (Cat# RG089M, Beyotime) on FlexStation^®^ 3 (Molecular Devices, San Jose, CA, USA).

### 4.13. Electrophoretic Mobility Shift Assay

An electrophoretic mobility shift assay (EMSA) was performed to assess the DNA-binding activity of the protein of interest. The binding sites of E2F1 in the promoter region of miR-29a (2000 bp upper-stranded and 500 bp down-stranded) were predicted by Tomtom [72] in the MEME Suite [73] against the known binding motif of E2F1 in the JASPAR database [74]. Double-stranded DNA probes were inserted into the pBR322 vector between the XbaI and BamHI cutting sites. Oligonucleotide probes were designed and amplified from plasmids with PCR (Cat# K0172, Thermo Scientific™). E2F1 protein was purchased from AtaGenix (~40.9kDa, Cat# YA6315H, Wuhan, China). Binding reactions were carried out in a total volume of 10 μL, containing E2F1 protein (0, 0.2, 0.4, 0.8, 1.6, and 3.2 μM) and ~280 ng double-stranded DNA probes, as provided by Table 5, with the binding site and mutated binding site highlighted. The reaction mixture was incubated at 37 °C for 30 min and 4 °C for 10 min. After incubation, the reaction mixtures were loaded on a 6% non-denaturing polyacrylamide gel in 0.5× TAE buffer with 6× novel juice fluorescent nucleotide dye (Cat# P001S1, Solarbio, Beijing, China). The electrophoresis was then carried out at 4 °C under a constant voltage of 80 V for 100 min. Bands corresponding to DNA–protein complexes were detected under UV light (BG-gdsAUTO 550, Baygene, Beijing, China).

### 4.14. Statistical Analysis

Data are presented as mean ± standard deviation (SD). Statistical comparisons among groups were performed using One-way ANOVA. Significance levels were defined as * *p* < 0.05, ** *p* < 0.01, *** *p* < 0.001, and **** *p* < 0.0001. Analyses were conducted using GraphPad Prism 10.0. Other data were visualized by R packages ggplot2 [75] and circos [76].

## 5. Conclusions

In conclusion, our study demonstrates that the CXG solution exerts multi-faceted anti-glioblastoma effects by simultaneously inducing apoptosis through the hsa-miR-10a-5p/*BCL2L11*/*BCL2* axis, suppressing extracellular matrix organization, and disrupting lipid metabolism via the *SREBF1*/*E2F1*/hsa-miR-29a-3p regulatory network. By coordinately downregulating critical lipid metabolism genes and modulating key tumor-promoting miRNAs, the CXG solution effectively targets multiple survival pathways in GBM cells. These findings not only provide mechanistic insights into CX’s anti-tumor activity but also highlight the therapeutic potential of combining miRNA-targeted approaches with metabolic intervention for GBM treatment. To translate these findings, activate phytochemical–target–miRNA regulatory network construction, in vivo models’ verification, and combination therapeutic strategy avenues merit priority. Pursuing these focused lines of inquiry will clarify pharmacodynamics, optimize delivery across the blood–brain barrier, and accelerate the clinical development of CX-derived agents for glioblastoma.

## Figures and Tables

**Table 1:** UHPLC-MS reveals the main components in the CXG solution.

	MZ	RT	Signal/ Noise	Peak Area	Score	Library MZ
Senkyunolide A	193.1217	6.509	2762.79	4.67251 × 10^11^	92.5	193.1222
Ligustilide	191.1063	6.948	1306.16	3.69138 × 10^11^	92.8	191.1065
Bayogenin	511.3385	9.55	751.56	1,689,154,816	85.5	511.34
Levistilide A	381.2047	7.872	632.55	61,290,749,952	90.7	381.2061
D-(+)-Pipecolinic acid	130.0862	0.809	612.75	5,109,982,720	85.1	130.0863
Nicotinic acid	124.0392	1.123	484.63	4,147,505,920	96.2	124.0393
Pipecolic acid	130.0862	1.114	285.69	2,745,387,264	89.9	130.0863
4-Guanidinobutyric acid	146.0921	0.8	241.74	3,073,896,192	97.2	146.0924
1-Ethoxynaphthalene	173.0958	6.581	217.18	7,683,143,680	82.6	173.0961
trans-3-Indoleacrylic acid	188.07	3.127	194.89	6,711,983,104	92.4	188.0706
alpha-Linolenic acid	279.2308	6.59	637.1	7,82,151,296	85.6	279.232
Neochlorogenic acid	355.101	3.407	88.53	3,302,652,416	94.4	355.1023
Ferulic acid	195.0649	4.262	57.12	4,942,265,856	95	195.0651
Cucurbitacin IIb	543.3287	7.899	54.43	305,734,048	83.9	543.33

**Table 2:** HPLC–UV absorption characteristics and molar concentrations of the main compounds in CXG solution.

Compound	Molecular Weight	Retention Time (min)	Compound Contents in Samples (mg/g)	CXG Solution Concentration	Molar Concentration (μM)
Ferulic acid	194.18	20.073	0–0.104	CH	185.345–198.667
				CM	133.802–143.42
				CL	80.277–86.047
Senkyunolide I	224.25	25.541	2.292–2.457	CH	139.603–155.777
				CM	100.781–112.457
				CL	60.465–67.470
Senkyunolide H	224.25	26.736	0.072–0.093	CH	10.712–25.189
				CM	7.733–18.184
				CL	4.64–10.91
Senkyunolide A	192.25	45.449	1.994–2.225	CH	43.584–47.993
				CM	31.464–34.647
				CL	18.877–20.787
Ligustilide	190.24	48.221	0.153–0.360	CH	0–8.592
				CM	0–6.202
				CL	0–3.721
Levistilide A	380.5	55.354	0.534–0.588	CH	2.988–3.821
				CM	2.157–2.759
				CL	1.294–1.655

**Table 3:** Concentration equivalent of the CXG solution and crude CX.

	CXG Solution Concentration (mg/mL)	CX Concentration (mg/mL)
CH	15.7	72
CM	11.334	52.1
CL	6.8	31.2

**Table 4:** Primers used for qPCR validation.

Gene/miRNA	Forward Primer (5′-3′)	Reverse Primer (5′-3′)
*GAPDH*	ACCCACTCCTCCACCTTTGAC	TGTTGCTGTAGCCAAATTCGTT
*SREBF1*	GCCCCTGTAACGACCACTG	CAGCGAGTCTGCCTTGATG
*E2F1*	CATCCCAGGAGGTCACTTCTG	GACAACAGCGGTTCTTGCTC
*BCL2L11*	TAAGTTCTGAGTGTGACCGAGA	GCTCTGTCTGTAGGGAGGTAGG
*BCL2*	GGTGGGGTCATGTGTGTGG	CGGTTCAGGTACTCAGTCATCC
*U6*	CGCTTCACGAATTTGCGTGTCAT	GCTTCGGCAGCACATATACTAAAAT
hsa-miR-10a-5p	GCAGTACCCTGTAGATCCGA	GGTCCAGTTTTTTTTTTTTTTTCAC
hsa-miR-29a-3p	CGCAGTAGCACCATCTGA	TCCAGTTTTTTTTTTTTTTTAACCGA

**Table 5:** Sequence of double-stranded DNA probes. Bold text indicates the positions of (mutated) E2F1 binding sites.

Probe Name	Probe Sequence (5′-3′)	Probe Name
hsa-miR-10a promoter region probe	AGGGACGACAACAGGCCACCTAATAAGATGATACCAAGAACTATACCATG**ACGGCGAA**AAAGAACTGCTGCAAAAAATAATTTGCCAAGGAGAACTCAGAGTACAAA	hsa-miR-10a promoter region probe
hsa-miR-10a promoter region mutated probe	AGGGACGACAACAGGCCACCTAATAAGATGATACCAAGAACTATACCATG**TTTTTTTTT**AAGAACTGCTGCAAAAAATAATTTGCCAAGGAGAACTCAGAGTACAAAGC	hsa-miR-10a promoter region mutated probe
positive control probe	CTTCAGCAAATACTGCGCGCTGACTCTTAAGGACTAG**TTTCGCGC**CCTTTCTCAAATTTAAGCGCGAAAACTACGTCATCTCCAGCGGCCACACCCGGCG	positive control probe

## Data Availability

The data presented in this study are openly available in the Sequence Read Archive at PRJNA1260138.

## Supplementary Materials

The following supporting information can be downloaded at: https://www.mdpi.com/article/10.3390/ph18101553/s1. Figure S1: UPHPLC-MS profile of the CXG solution, showing four major components in CXG; Figure S2: Differentially expressed genes regulated by CXG and their functional enrichment analysis. (A) Venn diagram of upregulated differentially expressed genes (DEGs) across CL, CM, and CH treatment groups compared with control. (B) Venn diagram of downregulated DEGs across the same comparisons. (C) MetaCore pathway enrichment analysis of all DEGs, with the top significantly enriched pathways (ranked by −log10 *p*-value) shown. Figure S3: Survival analysis shows that the lower expression of genes involved in extracellular matrix organization and lipid metabolism improves overall survival. Figure S4: Raman single peak intensity under different conditions. Four treatment conditions are shown from left to right: 0 (untreated control), CL (CXG low dose), CM (CXG medium dose), and CH (CXG high dose; doses as defined in Methods). For each condition, three rows are provided: White (top row): Bright-field micrographs. The red rectangle marks the region of interest that was raster-scanned for Raman mapping. Scale bar: 20 µm. Raman (middle row): Representative Raman mapping images reconstructed from integrated spectral intensity. These maps show the overall signal distribution and scanned area, confirming that Raman spectra were acquired across the full cell body. The consistent intensity range (1.6 × 10^4^–1.9 × 10^4^ a.u.) ensures comparability between treatments. Merge (bottom row): Composite overlays to visualize single-band maps of selected characteristic spectral bands and assign pseudo-colors, where different biochemical components can be visualized simultaneously within the same cell. Figure S5: Bipartite compound–target interaction network after filtering direct E2F1 modulators. Table S1: A list of CTI pairs collected from databases. Raw reads of both RNA and small RNA sequencing data were deposited on the Sequence Read Archive (SRA) with accession number PRJNA1260138.

## Abbreviations

The following abbreviations are used in this manuscript:

ADAMTSL1	A disintegrin and metalloproteinase with thrombospondin motifs like 1 (gene)
AEBP1	Adipocyte enhancer binding protein 1
BCL2	B-cell lymphoma 2 (gene)
BCL2L11	BCL2-like 11 (gene)
CX	Ligusticum Chuanxiong Hort.
CXG	CX dispensing granules
E2F1	Early region two binding factor transcription factor 1 (gene, protein)
ECM	Extracellular matrix
EGFR	Epithelial growth factor receptor (gene)
FN1	Fibronectin 1 (gene)
GAPDH	Glyceraldehyde-3-phosphate dehydrogenase (gene)
GBM	Glioblastoma
GO	Gene Ontology
HMGA2	High mobility group AT-hook 2 (gene)
HOXD10	Homeobox D10 (gene)
IC	Inhibitory concentration
LC	Liquid chromatography
LDA	Linear discriminant analysis
MAPK	Mitogen-activated protein kinase
miRNA	microRNA
MMP-14	Matrix metalloproteinase-14
MS	Mass spectrometry
Nur77	Nuclear receptor 4A1 (gene)
PI3K/Akt	Phosphoinositide 3-kinase/Protein kinase B
PLAUR	Plasminogen activator, urokinase receptor (gene)
PXN	Paxillin (gene)
qPCR	Quantitative polymerase chain reaction
RhoC	Ras homolog family member C (gene)
SCAP	SREBF chaperone
SREBF1	Sterol regulatory element-binding factor 1 (gene)
SREBP1	Sterol regulatory element-binding protein 1
TCM	Traditional Chinese medicine
TGM2	Transglutaminase 2 (gene)
U6	U6 small nuclear RNA (gene)
UHPLC	Ultra-high-performance liquid chromatography
uPAR	Urokinase receptor
