# Supplementary figures and images for: Ligusticum chuanxiong Hort. Targets hsa-miR-10a-5p to Potentially Induce Apoptosis and Modulate Lipid Metabolism in Glioblastoma: A Natural-Product-Based Therapeutic Strategy
Source: Pharmaceuticals (Basel). 2025 Oct 15;18(10):1553. doi: 10.3390/ph18101553 (PMC12566676; doi:10.3390/ph18101553)

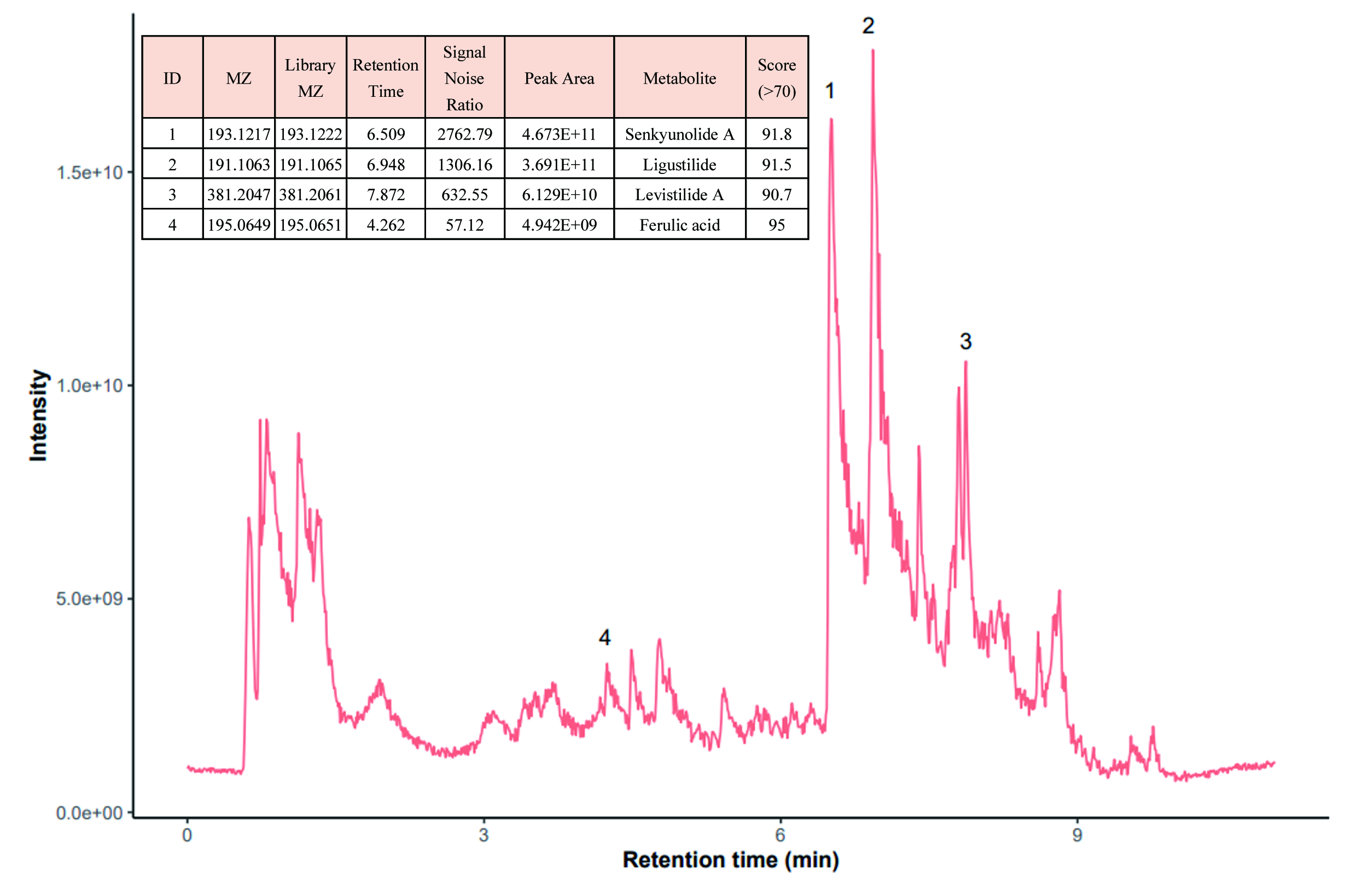

Supplement: Supplementary file 1 [file pharmaceuticals-18-01553-s001.zip › FigureS1.tif]

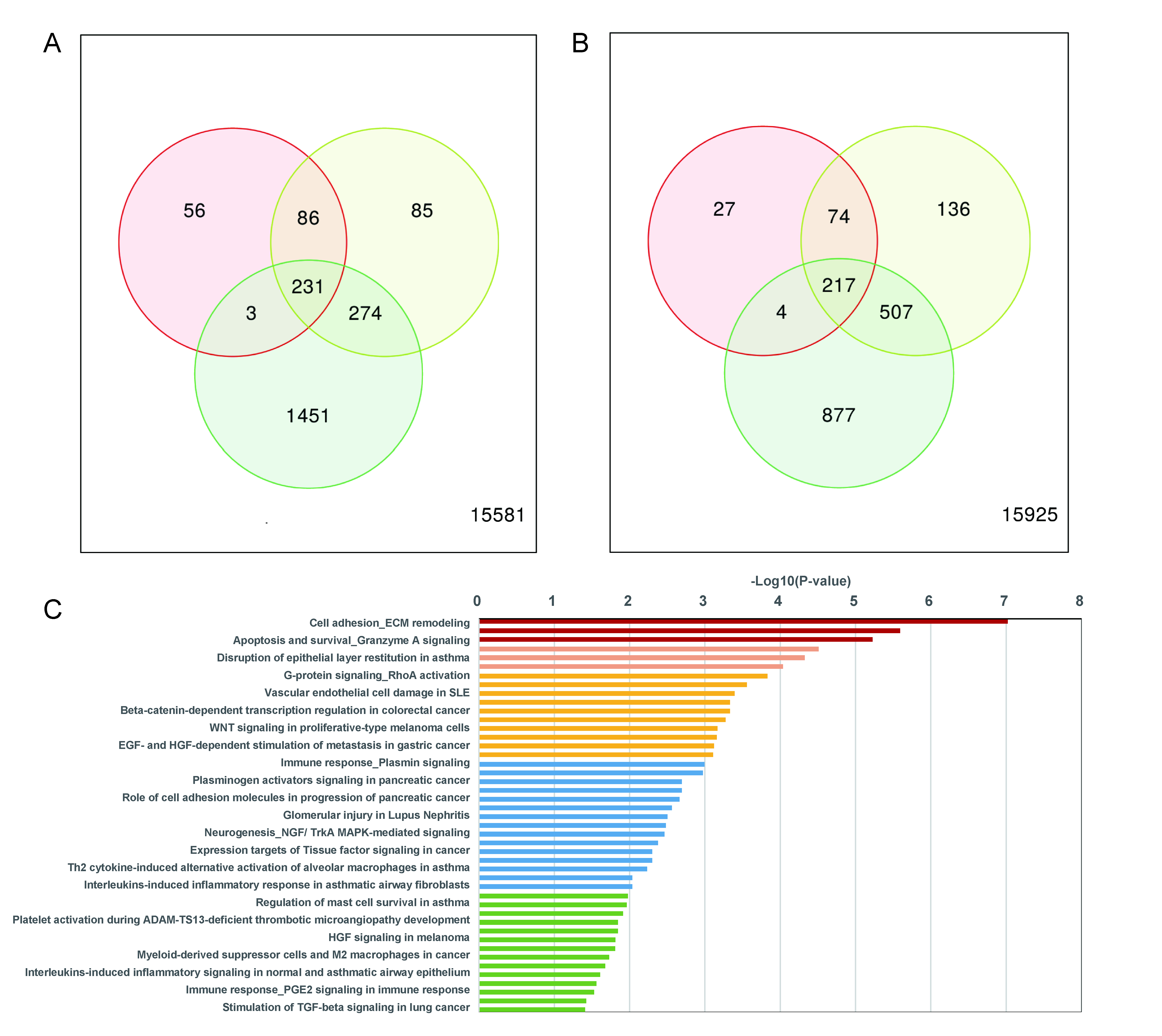

Supplement: Supplementary file 1 [file pharmaceuticals-18-01553-s001.zip › FigureS2.tif]

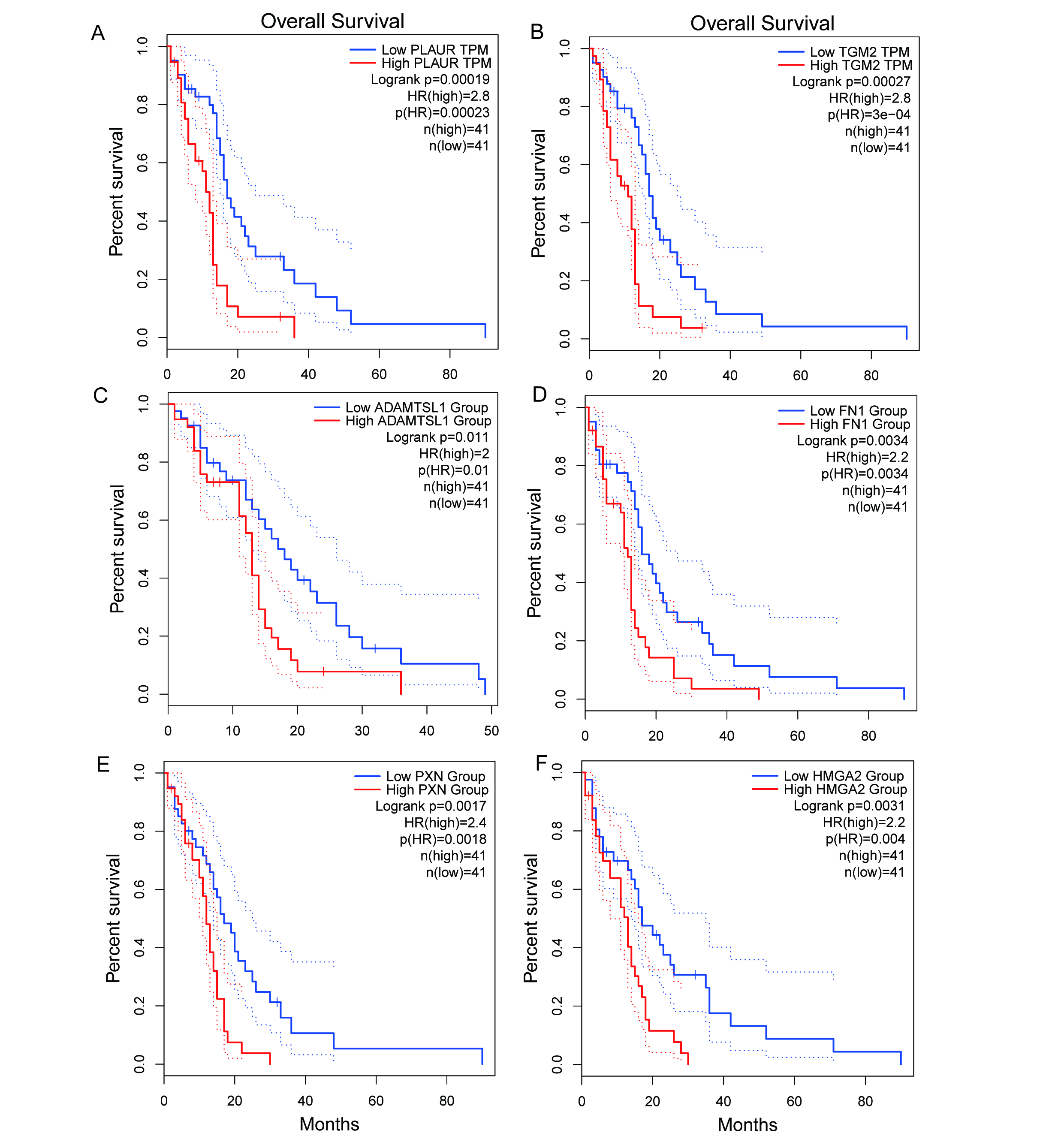

Supplement: Supplementary file 1 [file pharmaceuticals-18-01553-s001.zip › FigureS3.tif]

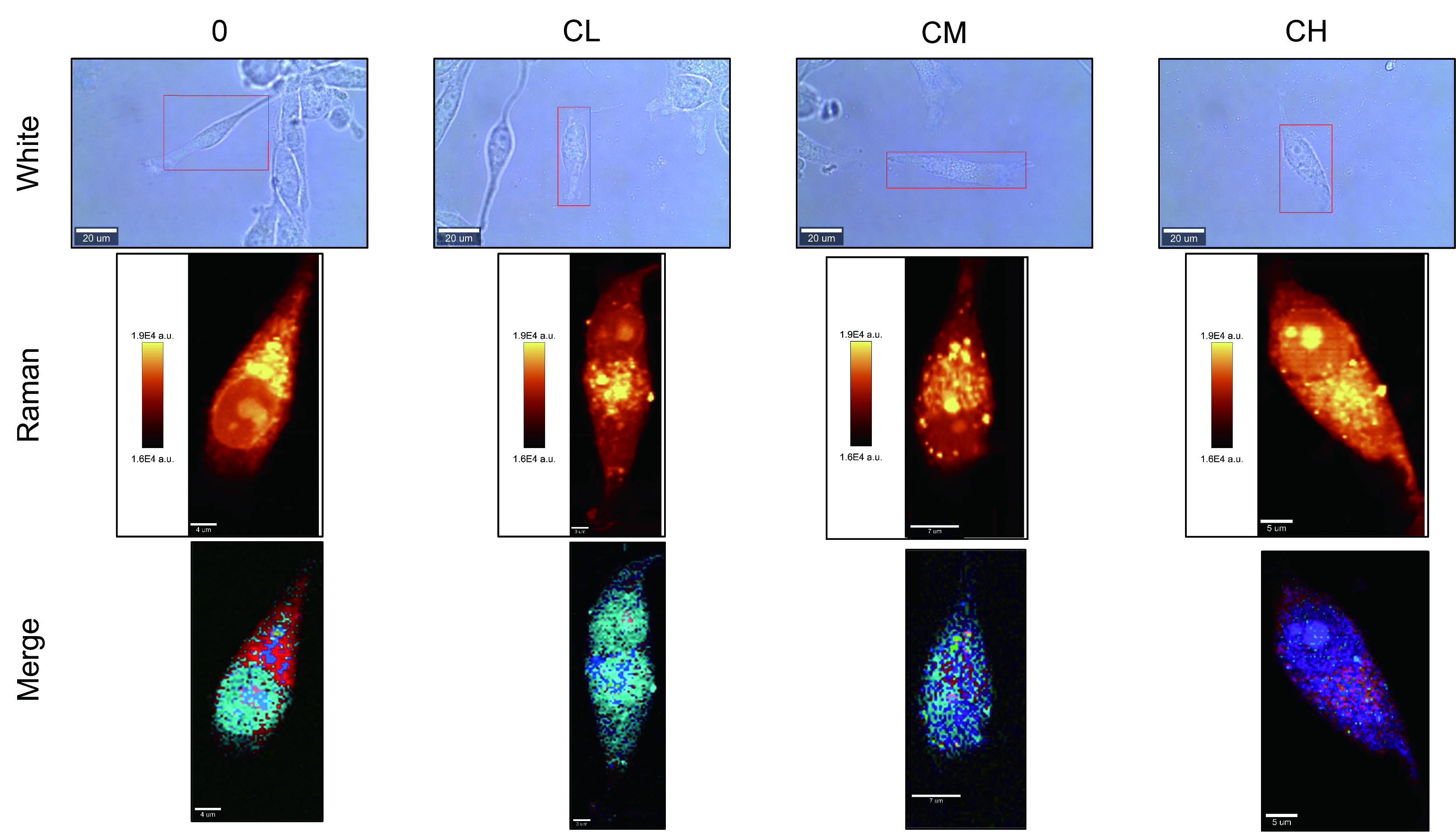

Supplement: Supplementary file 1 [file pharmaceuticals-18-01553-s001.zip › FigureS4.tif]

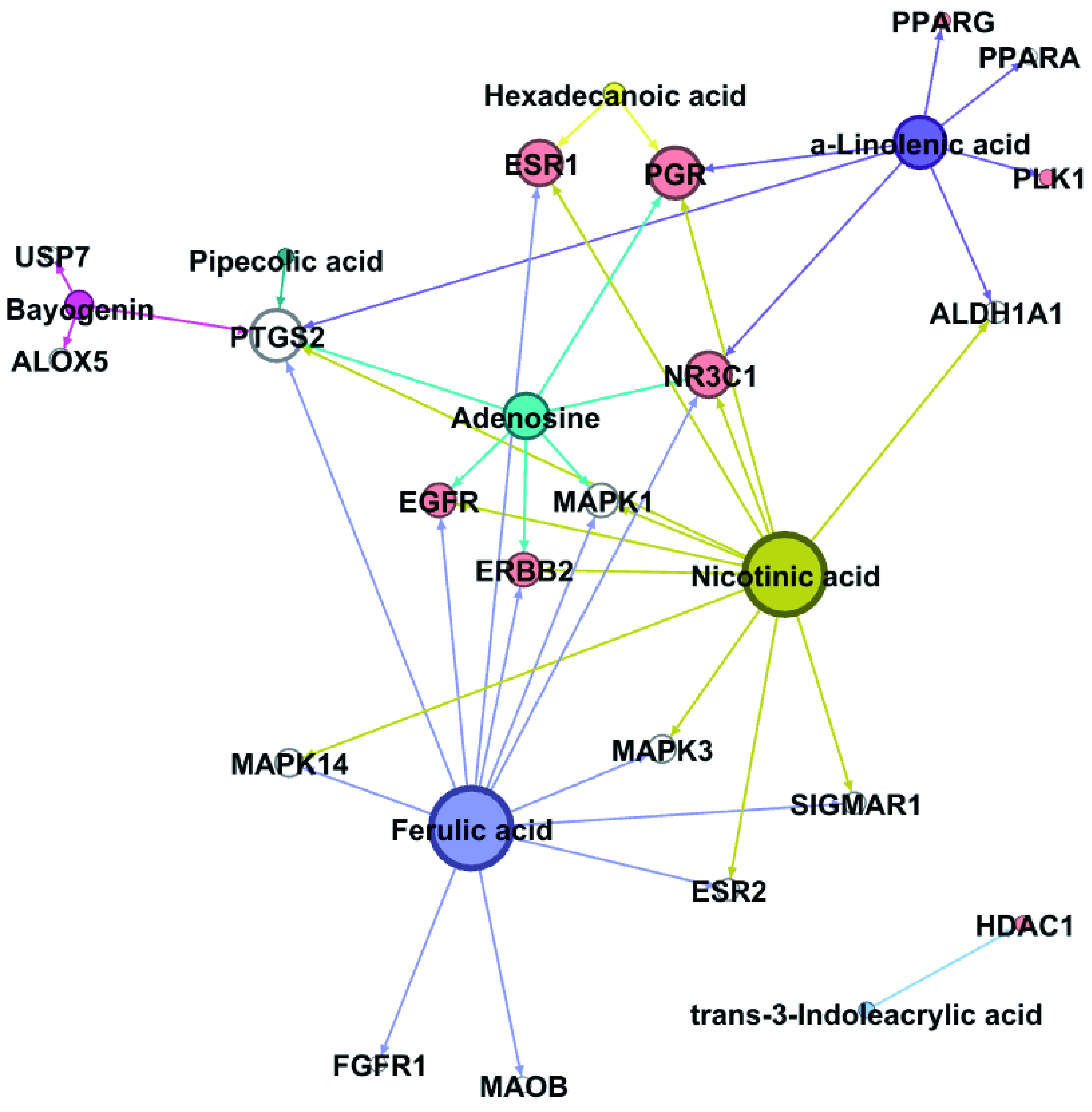

Supplement: Supplementary file 1 [file pharmaceuticals-18-01553-s001.zip › FigureS5.tif]
